# A fatal case of chronic *Cryptococcus neoformans* meningitis progressing over a 4-year period following cladribine therapy

**DOI:** 10.1128/asmcr.00061-24

**Published:** 2025-02-03

**Authors:** Christina Ekenberg, Jon Gitz Holler, Christian Peter Midtgaard Stenør, Karen Marie Thyssen Astvad, Jannik Helweg-Larsen, Micha Phill Grønholm Jepsen

**Affiliations:** 1 Department of Infectious Diseases, Copenhagen University Hospital, Rigshospitalet, Copenhagen, Denmark; 2 Department of Respiratory Medicine and Infectious Diseases, Copenhagen University Hospital, North Zealand, Hillerød, Denmark; 3 Department of Neurology, Copenhagen University Hospital, Herlev and Gentofte, Herlev, Denmark; 4 Unit of Mycology, Statens Serum Institut, Copenhagen, Denmark; Rush University Medical Center, Chicago, Illinois, USA

**Keywords:** cryptococcal meningitis, meningoencephalitis, cryptococcosis, *Cryptococcus neoformans*, chronic

## Abstract

**Background:**

Globally, cryptococcal meningitis (CM) remains most common in human immunodeficiency virus (HIV)-infected individuals. However, *Cryptococcus neoformans* increasingly causes CM in patients with non-HIV immunosuppression due to glucocorticoid treatment, organ transplantation, and hematological cancer, among others. The clinical presentations of cryptococcal disease are highly host dependent, resulting in varying disease presentations in immunocompromised patients.

**Case Summary:**

Here, we report a fatal case of CM in a patient previously treated with cladribine due to hairy cell leukemia. The patient had an atypical presentation, and the disease progressed slowly over a 4-year period, hampering timely diagnostics and treatment. Retrospective analyses of the cerebrospinal fluid and blood revealed that the cryptococcal antigen (CrAg) test was already positive when the patient initially presented with pleocytosis 3 years prior to the CM diagnosis.

**Conclusion:**

This case adds valuable knowledge to our current understanding of CM due to the unusual time course and furthermore demonstrates important pitfalls associated with cryptococcosis in HIV-negative patients, including the atypical disease presentation and diagnostic challenges which resulted in a diagnostic delay of 3 years. Morbidity and mortality remain high, and with a growing population of non-HIV immunocompromised patients, increased awareness of CM and low threshold to screen these patients for CrAg are warranted.

## INTRODUCTION

Meningitis is the most common manifestation of cryptococcosis and is predominantly observed in people with human immunodeficiency virus (HIV). In immunocompromised, HIV-negative patients, cryptococcal meningitis (CM)/cryptococcal meningoencephalitis is most often associated with severely reduced cellular immunity, particularly high-dose or prolonged corticosteroid therapy, organ transplantation, and hematological cancer (1–3). While HIV-associated CM tends to present itself with acute illness, the disease presentation is often more variable in patients with other forms of immune impairment, which may challenge accurate and timely diagnosis.

## CASE PRESENTATION

In April 2018, a previously healthy 51-year-old man was diagnosed with hairy cell leukemia (HCL) and treated with a 5-day course of cladribine. Subsequently, the patient was hospitalized due to neutropenia-related *Legionella pneumophila* and *Aspergillus fumigatus* pneumonia with septic shock. A computerized tomography (CT) scan revealed cavitating pulmonary infiltrates. Apart from aspergillus, no other infections were detected by bronchoscopy with biopsy and lavage. Follow-up scans showed resolving lung infiltrates.

**Fig 1:**
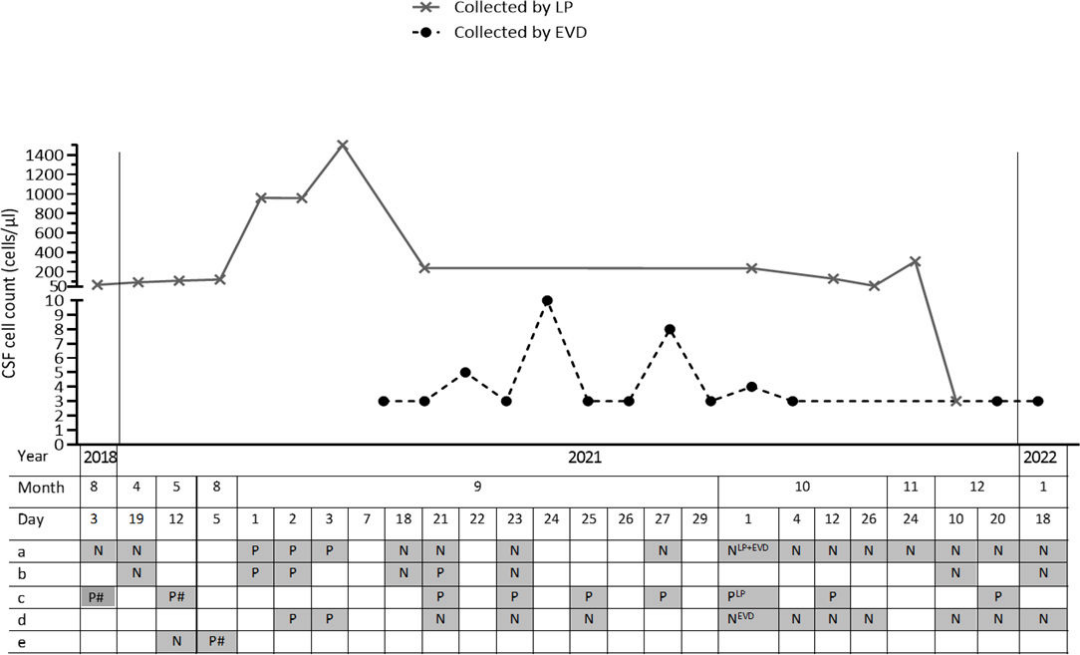
CSF examination (selected analyses). CSF collection dates (*x*-axis) and the corresponding CSF cell counts (*y*-axis). Microbiological analyses are indicated by a, b, c, d, and e, showing (a) culture; (b) multiplex NAT covering enterovirus, human parechovirus, HSV1, HSV2, cytomegalovirus, human herpesvirus 6, VZV, *Escherichia coli* (K1), *Haemophilus influenzae*, *Listeria monocytogenes*, *Neisseria meningitidis*, *Streptococcus agalactiae* (gr. B), *Streptococcus pneumoniae*, and *Cryptococcus neoformans*/*Cryptococcus gattii*; (c) cryptococcal antigen*; (d) microscopy following Alcian blue staining; and (e) microbiome test, respectively. Analyses are marked with P if positive, and N if negative. Analyses performed retrospectively are marked with #. Gray boxes denote analysis done, and white boxes denote analysis not carried out. Abbreviations: CSF, cerebrospinal fluid; EVD, external ventricular drain; HSV1, herpes simplex virus 1; HSV2, herpes simplex virus 2; LP, lumbar puncture; VZV, varicella zoster virus. *Upon diagnosis in August 2021 and throughout 2021, the Dynamiker Cryptococcal Antigen Lateral Flow Assay (Dynamiker Biotechnology [Tianjin] Co., Ltd.) was used, whereas retrospective analyses on blood and CSF samples from August 2018 and May 2021 were performed using Cryptococcal Antigen Latex Agglutination System (Meridian Bioscience).

In August 2018, the patient was readmitted with fever, headache, and vomiting. Meningitis was suspected and cerebrospinal fluid (CSF) was obtained, showing mixed pleocytosis (68 cells/µL) (Fig. 1), elevated protein (2.1 g/L), and a low CSF:blood glucose ratio (0.3). However, no infectious agents were detected by culture or PCR, and the patient recovered fully following a week of empiric antibacterial and antiviral therapy. He was in good clinical condition, attending regular checkups at the hematology outpatient clinic.

In April 2021, the patient was referred due to 6 months of relapsing headache and vertigo, and 2 months of binocular diplopia without fever. At admission, he had bilateral abducens nerve palsy. C-reactive protein (CRP), hematology, liver and kidney parameters were normal. Magnetic resonance imaging of the brain (MRI-brain) demonstrated diffuse leptomeningeal enhancement (LE) and enlarged ventricles. LP revealed CSF with mononuclear pleocytosis (93 cells/µL), elevated protein (3.1 g/L), and low CSF:blood glucose ratio (0.2). CSF investigations, including culture, multiplex nucleic acid test (BioFire FilmArray Meningitis-Encephalitis [FA/ME] Panel, BioMerieux), *Borrelia burgdorferi* intrathecal antibody production, flow cytometry and cytology, were negative. Relapse of HCL was suspected but ruled out, and the patient was treated empirically with 2 weeks of doxycycline.

Between April and August 2021, LP was repeated twice, revealing lymphocytic pleocytosis and elevated intracranial pressure (eICP) of 27 cm H_2_O. MRI-brain confirmed previous findings of hydrocephalus and LE. The patient was referred to neurosurgical biopsy, which was rejected due to a suspicion of carcinomatosis. Extensive microbiological testing, including CSF 16S and 18S amplicon-based next-generation sequencing (4) (microbiome test), was done without detection of infectious agents. A non-infectious chronic inflammatory meningitis was now suspected, and oral prednisone was initiated (25 mg increasing to 50 mg daily) with initial relief of symptoms.

At the end of August, after 4 weeks of corticosteroid treatment, the patient was admitted with headache, vomiting, and convulsive seizures. Fever and increased CRP ensued within 24 hours. CT of the brain revealed progression of hydrocephalus, and LP showed neutrophilic pleocytosis (960 cells/µL). FA/ME on CSF was positive for *Cryptococcus neoformans*/*Cryptococcus gattii*. The diagnosis was further confirmed by cryptococcal antigen (CrAg) (Dynamiker Cryptococcal Antigen Lateral Flow Assay, Dynamiker Biotechnology [Tianjin] Co., Ltd.) in blood, and cultures of blood and CSF yielded growth of *Cryptococcus neoformans* identified by matrix-assisted laser desorption ionization-time-of-flight mass spectrometry (Bruker, Bremen, Germany). Induction therapy with amphotericin B (3 mg/kg 1× daily) and 5-flucytosine (25 mg/kg 4× daily) was initiated. Blood samples showed a low CD4 T-cell count (67 cells/µL).

The eICP (48 cm H_2_O at admission) was initially managed by therapeutic LPs. However, due to persistent eICP, a lumbar drain, succeeded by an external ventricular drain (EVD) and ultimately a ventriculoperitoneal shunt, was placed. Two weeks following initiation of induction therapy, the patient was slowly recovering, and CSF collected from the EVD was normalized. A sudden impairment of the patient’s clinical condition prompted an MRI-brain, revealing thrombotic events and progression of the LE. To clarify the discrepancy between clinical and radiographic progression and normalization of CSF collected from the EVD, an LP was performed showing sustained pleocytosis (239 cells/µL).

Following 6 weeks of induction therapy and negative CSF cultures, treatment was switched to consolidation therapy with fluconazole. Clinically, the patient showed only minimal improvement, with fluctuating consciousness, hemiparesis, abducens nerve palsy, aphasia, and dysarthria. Magnetic resonance imaging (MRI) demonstrated increasing LE, arachnoiditis, widespread dural thickening, and gray matter alterations. Owing to these findings and lack of clinical improvement, post-infectious inflammatory response syndrome (PIIRS) was suspected. Adjunctive pulse corticosteroid therapy (PCT) (5) with intravenous methylprednisolone 1 g daily for 5 days, followed by tapered prednisone, was initiated. Despite a brief improvement, the patient’s condition overall remained unaltered. At the beginning of December 2021, due to worsening of MRI findings, PCT was repeated. Three weeks later, the patient suddenly deteriorated. MRI-neural axis revealed unaltered yet severe inflammatory changes, whereupon treatment with baricitinib was initiated. Despite attempts to reverse the patient’s PIIRS, an MRI-brain demonstrated end-stage inflammation with fibrosis. Baricitinib was discontinued, and corticosteroid tapering was initiated. Four days after cessation of baricitinib, the patient suddenly deteriorated and died.

Upon autopsy, histological examination of the brain showed numerous structures consistent with cryptococci throughout the meninges covering the brain stem, cerebellum, and hypothalamus, as well as ventriculitis with subependymal granulation. Retrospective analyses on frozen samples from May 2021 resulted in a positive CrAg (Cryptococcal Antigen Latex Agglutination System, Meridian Bioscience) in both CSF and blood (low [1:2] and high [1:8] titers, respectively), and microbiome test from August 2021 was positive for *Cryptococcus neoformans* DNA. Following approval from the Danish Research Ethics Committee (H-23043122), additional retrospective analyses were performed on blood and CSF samples collected in August 2018, revealing positive CrAg titers in both CSF (1:4) and blood (1:8).

## DISCUSSION

To the best of our knowledge, we present the first case of chronic CM spanning over 4 years. While cryptococcosis remains an HIV-associated disease globally, patients presenting with cryptococcal disease in high-income countries constitute a more heterogenous group (1, 6). Mortality remains high and seems to be higher in HIV-negative individuals (1), which is probably caused by multiple factors, including diagnostic delay due to atypical presentations and more comorbidities.

The patient presented with pleocytosis of unknown origin 3 months following cladribine treatment. Three years later, a similar pattern of pleocytosis was found. Initial CSF FA/ME and microbiome test results were negative, and CrAg was not analyzed. However, retrospective analyses performed on preserved CSF and blood revealed a positive CrAg as early as August 2018.

Previous case reports described a case of CM 19 months after cladribine therapy in a patient with HCL (7) and chronic relapsing CM in a patient with low mannose-binding lectin and a low naïve CD4 cell count (8), both exhibiting similarities with the present case. T-cell defects are highly associated with susceptibility to cryptococcosis (9). While being an effective first-line therapy for HCL, cladribine is also known to potentially induce prolonged CD4 lymphocytopenia (10), which may have been a contributing factor. Moreover, since infection was thought to be ruled out, corticosteroids were started on suspicion of non-infectious meningitis, which caused the fulminant CM.

Diagnosis was hampered by the slow progression, combined with a negative FA/ME and microbiome test and lack of CrAg test. Following his first admission with pleocytosis, the patient did not express symptoms for 2 years. Whether this period represents underreporting of symptoms or a state of latency (11) is unknown. Upon admission in April 2021, the patient presented with headache and abnormal vision but no fever. The varying host-dependent clinical presentations of cryptococcosis are contributing factors to diagnostic delays (11). Furthermore, our case emphasizes the importance of gaining a full overview of risk factors and knowledge about the limitations of diagnostic tests. While the FA/ME represents a powerful test in diagnosing meningitis in general, its use as a diagnostic test for CM remains debatable. Several studies have demonstrated its limitations in diagnosing CM due to poor sensitivity at lower fungal burdens (12, 13), which is often the case in HIV-negative patients (2).

The patient received PCT (5) for CM PIIRS (9). While a clinical benefit of PCT in CM PIIRS has been suggested previously (5), our case had a fatal outcome, and the reasons for this remain elusive. However, we did experience discrepancy between CSF collected through LP and EVD. This inconsistency was probably caused by severe ependymitis resulting in CSF compartmentalization due to blockage from the lateral and third ventricles to the fourth ventricle, potentially hampering complete fungal clearance. An endoscopic ventriculostomy might have relieved this obstruction and improved outcome.

In conclusion, this case demonstrates an atypical CM disease course and highlights the diagnostic and therapeutic challenges of CM, which are further challenged by the increasing variability of host risk factors. Diagnostic delays result in higher mortality (11). Hence, health-care professionals should have a low threshold for suspecting CM and screening for CrAg in immunocompromised patients presenting with pleocytosis. In this case, a CrAg test performed at an earlier time point may have led to a very different outcome. Due to lower fungal burden in HIV-negative patients, FA/ME and other PCR-based tests should not stand alone in ruling out CM.
